# Hip-Spine Discordance in Bone Mineral Densities in Patients Undergoing Dual Energy X-Ray Absorptiometry Scan in a Tertiary Care Centre: A Descriptive Cross-sectional Study

**DOI:** 10.31729/jnma.7951

**Published:** 2023-06-30

**Authors:** Rajesh Kumar Chaudhary, Subhash Regmi, Niraj Shah, Bibek Banskota, Ram Krishna Barakoti, Ashok Kumar Banskota

**Affiliations:** 1 Department of Orthopedics, B&B Hospital Pvt. Ltd., Gwarko, Lalitpur, Nepal

**Keywords:** *bone density*, *osteoporosis*, *prevalence*

## Abstract

**Introduction:**

The discordance in the diagnosis of osteoporosis is characterized by the variation in bone mineral density measurements at different skeletal sites. The presence of discordance significantly alters the treatment plan as different treatment is required for different skeletal sites. The aim of this study was to find out the prevalence of hip-spine discordance in bone mineral densities in patients undergoing dual-energy x-ray absorptiometry scans for suspected osteoporosis.

**Methods:**

A descriptive cross-sectional study was conducted among patients undergoing dualenergy x-ray absorptiometry scans from 1 December 2020 to 30 October 2022. Ethical approval was taken from the Institutional Review Committee (Reference number: IRC-2020-11-18-08). Patients undergoing dual-energy x-ray absorptiometry scans for suspected osteoporosis were included. Patients aged less than 50 years, already diagnosed and under treatment for osteoporosis, and incomplete information about T-scores of hips and spine were excluded. Convenience sampling method was used. Point estimate and 95% Confidence Interval were calculated.

**Results:**

Among 1028 patients, 602 (58.56%) (55.55-61.57, 95% Confidence Interval) had discordance in hip and spine bone mineral densities. The majority of them, 570 (94.68%) were female and 32 (5.71%) were male. Major discordance was observed in 101 (16.77%) patients and minor discordance was observed in 501 (83.22%) patients.

**Conclusions:**

The prevalence of discordance in hip and spine bone mineral densities in patients undergoing dual-energy x-ray absorptiometry scans was higher than that reported in other similar studies done in similar settings.

## INTRODUCTION

The discordance in the diagnosis of osteoporosis is characterized as the variation in bone mineral density (BMD) measurements at different skeletal sites, for example, hip and spine, using dual-energy x-ray absorptiometry (DXA).^1-3^ Major discordance occurs when it is normal at one site and osteoporosis at another or vice versa. Minor discordance occurs when it is normal at one site and osteopenia at another or osteopenia at one site and osteoporosis at another or vice versa.^3^

The presence of discordance alters the treatment plan because different treatment is required for different skeletal sites.^4^ Thus, it is essential to identify the prevalence of discordance to formulate therapeutic guidelines for osteoporosis. However, there are few studies in the literature reporting prevalence of discordance in hip and spine BMDs.

The aim of this study was to find out the prevalence of hip-spine discordance in bone mineral densities in patients undergoing dual-energy x-ray absorptiometry scans for suspected osteoporosis in a tertiary care center.

## METHODS

This descriptive cross-sectional study was conducted among the patients undergoing DXA scan for suspected osteoporosis from 1 December 2020 to 30 October 2022. Ethical approval was taken from the Institutional Review Committee of B&B Hospital Pvt. Ltd., Gwarko, Lalitpur, Nepal (Reference number: IRC-2020-11-18-08). Written informed consent was obtained from all the patients to be included in the study. Patients undergoing DXA scans for suspected osteoporosis were included in the study. Patients aged less than 50 years, already diagnosed and under treatment for osteoporosis, and incomplete information about T scores of hips and spine were excluded from the study. Convenience sampling method was used. The sample size was calculated using the following formula:


$$\begin{array}{ll} \text{n} & ={\text{Z}}^{2}\times \frac{\text{p}\times \text{q}}{{\text{e}}^{\text{2}}}\\ & =1.96^{2}\times \frac{0.41\times 0.59}{0.03^{2}}\\ & =1023 \end{array}$$

Where,

n = minimum required sample size
Z = 1.96 at 95% Confidence Interval (CI)
p = prevalence of discordance observed in the previous study, 41%^3^
q = 1-p
e = margin of error, 3%

The minimum required sample size was 1023. however, the final sample size taken was 1028.

All patients underwent DXA scan of the hip and spine using Hologic DXA Discovery Wi (S/N 86682) model operator using reference values of a population of white males and females provided by bone mineral density in childhood study (BMDCS) and the National Health and Nutrition Examination Surveys (NHANES). DXA scan was operated by a trained operator with more than five years of experience.

The DXA reports were categorized into two major categories: concordance and discordance. Concordance has three types: normal hip and normal spine, osteopenic hip and osteopenic spine, and osteoporotic hip and osteoporotic spine. Discordance has six types grouped into two categories: minor discordance, which includes the normal hip and osteopenic spine, osteopenic hip and normal spine, osteopenichip and osteoporotic spine, and osteoporotic hip and osteopenic spine, and major discordance, which includes normal hip and osteoporotic spine and osteoporotic hip and a normal spine, based on World Health Organization guideline.^3,4^

Age of the patients were grouped into 6 categories: 50-59, 60-69, 70-79, 80-89, 90 or more, and based of body mass index (BMI) patients were grouped into 4 categories: underweight (BMI <18.5), normal (BMI 18.5 to <25), overweight (25 to <30) and obese (30 or higher) kg/m^2^.

Data were entered and analysed using Microsoft Excel. Point estimate and 95% CI were calculated.

## RESULTS

Among 1028 patients included in the study, 602 (58.56%) (55.55-61.57, 95% CI) patients had discordance in hip and spine BMDs. Out of 602 patients in whom discordance was seen, 32 (5.71%) were males and 570 (94.68%) were females. Major discordance was observed in 101 (16.77%) patients and minor discordance was observed in 501 (83.22%) patients (Table 1).

**Table 1:** Pattern of discordance in hip and spine BMDs (n= 602).

Patterns	Hip	Spine	n (%)
Major discordance	Normal	Osteoporosis	100 (16.61)
	Osteoporosis	Normal	1 (0.16)
	Total		101 (16.77)
	Normal	Osteopenia	188 (31.22)
Minor discordance	Osteopenia	Normal	16 (2.65)
	Osteopenia	Osteoporosis	288 (47.84)
	Osteoporosis	Osteopenia	9 (1.49)
	Total		501 (83.22)

Age group 50-59 years had maximum discordance 243 (40.36%) and 260 (43.18%) had normal BMI (Table 2).

**Table 2:** Discordance according to age and BMI (n= 602).

Parameters		n (%)
Age (in years)	50-59	243 (40.36)
	60-69	209 (34.71)
	70-79	124 (20.59)
	80-89	24 (3.98)
	90 or more	2 (0.33)
BMI (kg/m^2^)	Underweight	7 (1.16)
	Normal	260 (43.18)
	Overweight	221 (36.71)
	Obese	114 (18.93)

## DISCUSSION

In this study, the prevalence of discordance in hip and spine BMDs in patients undergoing DXA scan was 602 (58.56%) among 1028 patients. The findings were higher compared to what was reported in studies conducted in Iran, United States Pakistan, India, and France.^3-7^ A study conducted in the United States evaluated DXA scan reports of 5051 female patients and observed discordance of 44% in hip and spine BMDs.^4^ Another study conducted in Iran involving 4188 participants observed discordance of 41.6% in hip and spine BMDs.^3^ Similarly, studies conducted in India and Pakistan observed discordance of 51.15% (involving 380 postmenopausal women) and 43.5% (involving 692 participants), respectively.^5,6^ Furthermore, a study conducted in France involving 1780 participants observed discordance of 50.5%.^7^ This suggested that discordance in hip and spine BMDs can be present in around 41-52% of the population undergoing DXA scans for suspected osteoporosis. The exact reason behind slightly higher discordant BMDs in our study is not clear. However, this could be due to the inclusion of a higher number of females aged more than 50. This argument is well supported by a study conducted in Morocco, in which they conducted found that age of more than 65 years is the single most important risk factor for discordant BMDs.

In this study, major discordance in hip and spine BMDs was observed in 9.82% of the patients and the majority of them had a normal hip and osteoporotic spine. The finding was higher compared to what was reported in studies conducted in the United States, Iran, Pakistan, and France, which was around 2.7%-5%.^4,3,5,7^ However, these studies included participants of both sexes. In addition, the population of western geographical areas is found to have higher BMDs than that of healthy individuals of Indian origin.^8^ Furthermore, it is also known that the spines are usually affected more than the hip in cases of secondary osteoporosis resulting in major discordance in BMD measurements.^9^ Contrastingly, The study conducted in India found a major discordance of 16.67% among 348 post-menopausal women.^6^ This suggests that the prevalence of major discordance in hip and spine BMDs can be higher, especially in post-menopausal women of the Indian subcontinent.

Similarly, in this study, minor discordance in hip and spine BMDs were observed in 48.73% of the patients. The findings were slightly higher compared to the findings reported by studies conducted in Iran, United States, Pakistan, India, and France which was around 35-46%.^3-7^ Several factors may have contributed to minor discordance in hip and spine BMDs, such as artifacts, technical measurements, using reference values of white healthy males and females, and pathophysiological causes.^2,4^

All patients undergoing DXA scans for suspected osteoporosis were included which could include a wide range of participants having several confounding factors that can influence T-score values, such as contraceptive use, duration of menopause, hormonal therapies, vitamin D supplementation therapy, and activity status.^1,10,11^ The outcomes were compared with the prevalence of discordance obtained in the international studies, which may have considerable variations in the selection of participants, their nutritional and activity status, other co-morbidities and risk factors for the change in BMDs, and usage of reference values for T score measurements. The outcomes of this study can help in understanding the pattern of discordance in the local population and help clinicians to propagate site-specific management of osteoporosis and fracture prevention.

This study has some limitations. This is a Cross-sectional study that has its own inherent study design-related limitations, such as selection and reporting bias.

## CONCLUSIONS

The prevalence of discordance in hip and spine BMDs in patients undergoing DXA scans for suspected osteoporosis was higher than that reported in other international studies.
